# Association of Body Mass Index and Mortality in Japanese Diabetic Men and Women Based on Self-Reports: The Japan Collaborative Cohort (JACC) Study

**DOI:** 10.2188/jea.JE20150011

**Published:** 2015-08-05

**Authors:** Yasuhiko Kubota, Hiroyasu Iso, Akiko Tamakoshi

**Affiliations:** 1 Public Health, Department of Social Medicine, Osaka University Graduate School of Medicine, Suita, Osaka, Japan; 1 大阪大学大学院医学系研究科社会医学公衆衛生学教室; 2 Department of Public Health, Hokkaido University Graduate School of Medicine, Sapporo, Japan; 2 北海道大学大学院医学系研究科社会医学講座公衆衛生学分野

**Keywords:** diabetes, body mass index, mortality, infection, obesity paradox

## Abstract

**Background:**

The association between body mass index (BMI) and mortality among Asian diabetic people, especially with respect to the obesity paradox (ie, higher BMI is associated with lower mortality risk), remains unresolved.

**Methods:**

We followed a cohort of 3851 self-reported Japanese diabetics (2115 men and 1736 women) in the Japan Collaborative Cohort Study from 1988–1990 through 2009. Individuals were aged 40 to 79 years and free from a history of cardiovascular disease, cancer, renal disease, or tuberculosis. BMI was grouped into the following four categories: <20.0, 20.0–22.9, 23.0–24.9, and ≥25.0 kg/m^2^.

**Results:**

During 54 707 person-years of follow-up, 1457 deaths from all causes, 445 from cardiovascular disease, 421 from cancer, 43 from renal disease, and 148 from infectious disease were documented. Mortality from all causes, cardiovascular disease, cancer, and renal disease showed L-shaped associations with BMI. Compared to diabetics with BMI of 20.0–22.9 kg/m^2^, those with BMIs of 23.0–24.9 kg/m^2^ and ≥25.0 kg/m^2^ had lower risks of mortality from infectious disease (ie, obesity paradox). The multivariable HRs for mortality from infectious disease were 0.50 (95% confidence interval, 0.31–0.81) and 0.51 (95% confidence interval, 0.32–0.82) among participants with BMIs of 23.0–24.9 kg/m^2^ and ≥25.0 kg/m^2^, respectively. Similar results were observed after stratification by smoking status and age and exclusion of early deaths.

**Conclusions:**

We observed L-shaped associations between BMI and mortality from all causes, cardiovascular disease, cancer, and renal disease, while the association between BMI and mortality from infectious disease manifested the obesity paradox.

## 要約

**背景：:**

アジアの糖尿病患者におけるbody mass index (BMI) と死亡との関連、特に、肥満パラドックス（BMIが大きくなるにつれ死亡リスクが低下する）が存在するかどうかに関しては解決していない。

**方法：:**

JACC studyで自己申告による40-79歳の糖尿病患者のうち、循環器疾患、がん、腎臓病、および結核の既往のない3,851人（男性2,115人、女性1,736人）を1998-1990年から2009年まで追跡した。BMIで<20.0、20.0–22.9（reference）、23.0–24.9、および≥25.0kg/m2の4群に分類し、Cox比例ハザードモデルを用いて死亡リスクを比較した。

**結果：:**

54,707人年の追跡期間中、全死亡1,457件、循環器死亡445件、がん死亡421件、腎不全死亡43件、および感染症死亡148件を認めた。全死亡、循環器死亡、がん死亡、および腎不全死亡のリスクとBMIとの関連はL字型を示した。一方、感染症死亡リスクとBMIと関連に関しては、BMI 20.0–22.9kg/m^2^に比べて、 BMI 23.0–24.9、≥25.0kg/m^2^の群は死亡リスクが低下する傾向にあった（肥満パラドックス）。多変量解析の結果、有意に感染症死亡リスクが低下していたのは、 BMI 23.0–24.9kg/m^2^でハザードリスク（95%信頼区間）=0.50 (0.31-0.81)、およびBMI 25.0–27.4kg/m^2^で0.51 (0.32–0.82)であった。喫煙状況および年齢で層化、また、早期死亡を除外した後も感染症死亡リスクとBMIとの関連は同様であった。

**結語：:**

全死亡、循環器死亡、がん死亡、および腎不全死亡リスクとBMIとの関連はL字型を示したが、感染症死亡リスクとBMIとの関連では肥満パラドックスを認めた。

## INTRODUCTION

In Western countries, vigorous discussions surround the association between body mass index (BMI) and mortality among diabetic people. Specifically, certain conclusions have been reached with respect to the “obesity paradox”—the observed phenomenon of lower mortality among overweight or obese diabetic people compared with normal-weight diabetic people.^1^^–^^5^ On the other hand, in Asia, the evidence for this association is limited.^6^^–^^8^ Since Asian diabetic people are unique in that they are likely to be leaner than Western diabetic people, in part because of genetic differences,^9^^,^^10^ it is necessary to obtain more data on the association between BMI and mortality among Japanese diabetic people.

Here, we conducted a prospective cohort study to analyze the association of BMI with mortality among Japanese diabetic people. The current study set as endpoints not only all-cause but also diabetes-related cause-specific mortality, including death from cardiovascular disease, cancer, renal disease, and infectious disease.^11^ This broad view should cultivate our understanding of the above-mentioned association because overweight or obese diabetic people, who usually have better nutritional status, might have lower risks of mortality from some kinds of diseases, such as cancer and infectious disease, than their under- or normal-weight equivalents. In addition, when investigating the obesity paradox, we must consider the influence of smoking, age, and underlying frailty, because these factors can facilitate both weight loss and mortality risk. Therefore, observations of the obesity paradox in the context of mortality from certain diseases require further analyses by stratification of participants according to smoking status and age (older and younger age) and exclusion of early deaths. These methodological considerations should provide us with more meaningful and accurate associations between BMI and mortality among diabetic people.

## METHODS

### Study population

The Japan Collaborative Cohort (JACC) Study for Evaluation of Cancer Risks, sponsored by the Ministry of Education, Culture, Sports, Science and Technology, conducted a baseline survey from 1988 to 1990 in 45 areas throughout Japan. Participants completed self-administered questionnaires concerning their lifestyle and medical history of cardiovascular disease and cancer. Details of this survey have been described previously.^12^ Participants comprised 110 585 individuals (46 395 men and 64 190 women) aged 40 to 79 years. Of these participants, 5283 self-reported diabetic subjects (2879 men and 2404 women) were selected. We excluded 309 subjects (130 men and 179 women) because of missing information on BMI, as well as 1123 subjects (634 men and 489 women) who had a history of cardiovascular disease, cancer, renal disease, or tuberculosis, due to the potential influence of these comorbidities on our outcomes. Consequently, 3851 subjects (2115 men and 1736 women) were included in the study. Self-reported diabetes is usually in agreement with reviews of the medical records and is believed to be highly specific but only moderately sensitive.^13^^,^^14^ A previous study showed high validity of self-reported diabetes gained from our questionnaire by comparing self-reported data with laboratory findings and treatment status in a sample of 1230 men and 1837 women as follows: the sensitivity of self-reporting was 70% for men and 75% for women; the specificity was 95% for men and 98% for women.^15^ Ethics committees of the Nagoya University School of Medicine and Osaka University Graduate School of Medicine approved the study.

### Mortality surveillance

Mortality surveillance was conducted systematically by reviewing death certificates, all of which were forwarded by the centers that serviced the people who had died. Mortality data were then centralized at the Ministry of Health and Welfare, and the underlying causes of death were coded for the National Vital Statistics according to the International Classification of Diseases, 10th Revision (ICD-10). Registration of death is required by the Family Registration Law in Japan. The participants were followed up until death, or to the end of 2009, except for four areas in 1999, four areas in 2003, and two areas in 2008, where the follow-up had been terminated. We used not only all-cause but also diabetes-related cause-specific mortality, including death from cardiovascular disease, cancer, renal disease, and infectious disease, as endpoints.^11^ Deaths from various diseases were defined by ICD-10 codes as follows: cardiovascular disease, I00 to I99; cancer, C00 to C97 or D00 to D48; renal disease, N00 to N08, N14, or N17 to N19; and infectious disease, A00 to A99, B00 to B99, G00 to G02, G09, I33, I38 to I41, I52, J00 to J22, J85 to J86, K65, K67, L00 to L08, M00 to M03, M86, M90, N10 to N13, N15, or N39. We referred to the methodology of a previous study to define deaths from infectious disease as above.^16^

### Main exposure: body mass index

Participants were requested to provide height and weight information. BMI was calculated as weight (kg)/height (m^2^). With reference to the World Health Organization (WHO) classification^17^ and our previous study,^18^ BMI was grouped into the following four categories: <20.0, 20.0–22.9 (reference), 23.0–24.9, and ≥25.0 kg/m^2^. In addition, as suggested by the WHO, BMIs of <18.5, 18.5–24.9, 25.0–29.9, and ≥30.0 kg/m^2^ were defined as underweight, normal weight, overweight, and obese, respectively.

### Potential confounding factors

A broad set of potential confounding factors were determined from the self-reported data at baseline. These included age, sex, history of hypertension (yes or no), drug treatment for diabetes (yes or no), family history of diabetes (yes or no), alcohol intake (never, past drinker, and current drinker with an ethanol intake of 1–22, 23–45, 46–48, or ≥49 grams per day), smoking status (never, past smoker, and current smoker of 1–19 or ≥20 cigarettes per day), perceived mental stress (low, medium, or high), walking (rarely, 30, 30–60, or ≥60 minutes per day), sports participation (rarely, 1–2, 3–4, or ≥5 hours per week), sleep duration per day (hours per day; continuous), and energy intake (kilocalories per day; quintiles).

### Statistical analysis

The person-years of follow-up for each participant were calculated from the baseline in 1988 to 1990 to the first endpoint: death, moving from the community, or the end of follow-up. We conducted pooled analyses of men and women because sex-specific analyses showed low statistical power. Age- and sex-adjusted mean values and the prevalence of selected factors were calculated and compared among the four groups using ANOVA and *r*^2^ tests, respectively. Using Cox proportional hazard models, we calculated the hazard ratios (HRs) and their 95% confidence intervals (CIs) for mortality outcomes with reference to the risks for BMI between 20.0–22.9 kg/m^2^, after adjustment for age, sex, and other potential confounding factors. SAS Version 9.3 software (SAS Institute Inc., Cary, NC, USA) was used for statistical analysis. All statistical tests were two-tailed, and *P* values of <0.05 were considered significant.

## RESULTS

The proportions of underweight (BMI <18.5 kg/m^2^), normal weight (18.5–24.9 kg/m^2^), overweight (25.0–29.9 kg/m^2^), and obese (≥30.0 kg/m^2^) people were 5.3%, 69.3%, 23.0%, and 2.4%, respectively. There were no diabetic people with BMI ≥40.0 kg/m^2^ (data not shown). Table 1 shows baseline characteristics according to BMI. Participants were more likely to be younger, female, and hypertensive as BMI increased. In addition, people with BMI 20.0–22.9 kg/m^2^ were more likely to walk for more than 30 minutes per day than those with other BMIs.

**Table 1.** Age- and sex-adjusted mean values and prevalence of baseline characteristics according to body mass index

Body mass index, kg/m^2^	<20.0 kg/m^2^	20.0–22.9 kg/m^2^ (reference)	23.0–24.9 kg/m^2^	≥25.0 kg/m^2^	*P* value
Number at risk	563	1418	893	977	
Age, years	63.2	60.9	60.6	59.5	<0.001
Male, %	59.3	58.2	56.0	46.7	<0.001
History of hypertension, %	24.0	34.2	42.1	47.2	<0.001
Drug treatment for diabetes, %	49.0	48.1	46.3	47.4	0.740
Family history of diabetes, %	15.9	13.4	12.3	11.1	0.134
Ethanol intake, grams/day	28.4	29.6	31.9	33.1	0.087
Current smoker, %	37.5	33.5	32.6	31.9	0.094
High perceived mental stress, %	24.6	23.9	20.5	21.9	0.286
Walking ≥30 minutes/day, %	84.9	89.4	84.7	85.1	0.009
Sports participation ≥1 hour/week, %	31.0	34.6	31.6	31.1	0.291
Sleep duration, hours/week	7.3	7.3	7.4	7.5	0.091
Energy intake, kilocalories/day	1439	1488	1498	1490	0.262

In the 54 707 person-years of follow-up of 3851 subjects (2115 men and 1736 women), we documented 1457 deaths from all causes (873 men and 584 women), 445 from cardiovascular disease (241 men and 204 women), 421 from cancer (284 men and 137 women), 43 from renal disease (22 men and 21 women), and 148 from infectious disease (93 men and 55 women) (Table 2). Age- and sex-adjusted HRs and multivariable HRs (95% CI) were calculated for not only all-cause mortality but also for mortality from cardiovascular disease, cancer, renal disease, and infectious disease according to BMI. Both age- and sex-adjusted HRs and multivariable HRs for mortality from all-cause, cardiovascular disease, cancer, and renal disease showed L-shaped relationships. The significant multivariable HRs were 1.35 (95% CI, 1.16–1.57) for all-cause mortality among diabetic people with BMI <20.0 kg/m^2^, 1.40 (95% CI, 1.05–1.85) for cardiovascular mortality among those with BMI <20.0 kg/m^2^, 1.33 (95% CI, 1.01–1.77) for cancer mortality among those with BMI <20.0 kg/m^2^, and 2.49 (95% CI, 1.03–6.02) for mortality from renal disease among those with BMI <20.0 kg/m^2^. In contrast, compared with diabetic people with BMI of 20.0–22.9 kg/m^2^, those with BMI of 23.0–24.9 kg/m^2^ and >25.0 kg/m^2^ had lower risks of mortality from infectious disease (demonstrating the obesity paradox). Significant multivariable HRs for mortality from infectious disease were 0.50 (95% CI, 0.31–0.81) and 0.51 (95% CI, 0.32–0.82) among diabetic people with BMIs of 23.0–24.9 kg/m^2^ and >25.0 kg/m^2^, respectively.

**Table 2.** Age- and sex-adjusted hazard ratios and multivariable^a^ hazard ratios for cause-specific mortality according to body mass index

Body mass index, kg/m^2^	<20.0 kg/m^2^	20.0–22.9 kg/m^2^ (reference)	23.0–24.9 kg/m^2^	≥25.0 kg/m^2^
Person-years at risk	7120	20 386	12 901	14 300
All-cause				
Total number of deaths	267	527	316	347
Age- and sex-adjusted HR (95% CI)	1.29 (1.11–1.49)	1.00	0.98 (0.85–1.12)	1.02 (0.89–1.17)
Multivariable HR (95% CI)	1.35 (1.16–1.57)	1.00	0.96 (0.83–1.11)	0.96 (0.83–1.10)
Cardiovascular disease				
Total number of deaths	77	147	104	117
Age- and sex-adjusted HR (95% CI)	1.30 (0.99–1.72)	1.00	1.15 (0.90–1.48)	1.22 (0.96–1.56)
Multivariable HR (95% CI)	1.40 (1.05–1.85)	1.00	1.11 (0.86–1.43)	1.10 (0.86–1.41)
Cancer				
Total number of deaths	75	148	104	94
Age- and sex-adjusted HR (95% CI)	1.33 (1.01–1.76)	1.00	1.14 (0.89–1.47)	1.00 (0.77–1.30)
Multivariable HR (95% CI)	1.33 (1.01–1.77)	1.00	1.14 (0.89–1.47)	0.99 (0.76–1.29)
Renal disease				
Total number of deaths	9	14	8	12
Age- and sex-adjusted HR (95% CI)	1.64 (0.71–3.80)	1.00	0.93 (0.39–2.22)	1.29 (0.60–2.80)
Multivariable HR (95% CI)	2.49 (1.03–6.02)	1.00	0.79 (0.32–1.95)	1.14 (0.51–2.56)
Infectious disease				
Total number of deaths	30	70	22	26
Age- and sex-adjusted HR (95% CI)	1.06 (0.69–1.63)	1.00	0.52 (0.32–0.84)	0.59 (0.38–0.93)
Multivariable HR (95% CI)	1.19 (0.77–1.85)	1.00	0.50 (0.30–0.81)	0.51 (0.32–0.82)

CI, confidence interval; HR, hazard ratio.

^a^Adjusted for age, sex, history of hypertension, drug treatment for diabetes, family history of diabetes, alcohol intake, smoking status, perceived mental stress, walking, sports, sleep duration, and energy intake.

In addition, in order to examine the influence of smoking, age, and underlying frailty on the association of BMI with mortality from infectious disease, we repeated our analyses by stratifying participants according to smoking status and age (≥65 years old and <65 years old) and excluding early deaths (Table 3). The multivariable HRs for mortality from infectious disease in each stratified group were lower for overweight and obese people (again demonstrating the obesity paradox).

**Table 3.** Multivariable^a^ hazard ratios for mortality from infectious disease according to body mass index, stratified by smoking status, age, and exclusion of early death

Body mass index, kg/m^2^	<20.0 kg/m^2^	20.0–22.9 kg/m^2^ (reference)	23.0–24.9 kg/m^2^	≥25.0 kg/m^2^
Never smoker				
Person-years at risk	2838	8744	5711	7215
Infectious disease, number of deaths	10	19	12	10
Multivariable HR (95% CI)	1.60 (0.70–3.66)	1.00	0.96 (0.45–2.04)	0.63 (0.28–1.41)
Current smoker				
Person-years at risk	2407	6255	3924	3558
Infectious disease, number of deaths	11	24	4	6
Multivariable HR (95% CI)	1.92 (0.87–4.23)	1.00	0.34 (0.11–1.02)	0.53 (0.20–1.37)
<65 years old				
Person-years at risk	4441	14 475	9253	10 236
Infectious disease, number of deaths	10	32	7	9
Multivariable HR (95% CI)	1.19 (0.57–2.48)	1.00	0.34 (0.15–0.79)	0.38 (0.18–0.81)
≥65 years old				
Person-years at risk	2679	5912	3649	4064
Infectious disease, number of deaths	20	38	15	17
Multivariable HR (95% CI)	1.25 (0.71–2.20)	1.00	0.55 (0.30–1.03)	0.54 (0.30–0.98)
Exclusion of those who died within 5 years				
Person-years at risk	6915	20 147	12 761	14 154
Infectious disease, number of deaths	23	63	17	24
Multivariable HR (95% CI)	1.02 (0.63–1.67)	1.00	0.42 (0.25–0.73)	0.51 (0.32–0.83)

CI, confidence interval; HR, hazard ratio.

^a^Adjusted for age, sex, history of hypertension, drug treatment for diabetes, family history of diabetes, alcohol intake, smoking status, perceived mental stress, walking, sports, sleep duration, and energy intake except for each stratified variable.

## DISCUSSION

In this prospective cohort study of Japanese diabetics, we observed that, compared with diabetic people with BMI of 20.0–22.9 kg/m^2^, diabetic people with higher BMIs had lower risks of mortality only from infectious disease, demonstrating the obesity paradox in this context, while those with lower BMIs had higher risks of mortality from all-cause, cardiovascular disease, cancer, and renal disease. To the best of our knowledge, this is the first study to investigate the association between BMI and diabetes-related cause-specific mortality in Japanese diabetics, shedding light on an iteration of the obesity paradox in mortality from infectious disease.

This paradox was maintained even after stratification by smoking status and age and exclusion of early deaths, as shown in Table 3. We speculate two hypotheses for the obesity paradox observed in this study. First, because excess weight generally reflects better nutritional status and greater energy reserves, overweight or obese diabetic people might be less likely to die from infectious disease than those of normal weight. Second, overweight or obese diabetic people might be more likely to die from cardiovascular disease and cancer than from infectious disease. In either case, however, it is still unknown whether extremely obese diabetic people with BMI ≥40.0 kg/m^2^ have lower risk of mortality from infectious disease, although we concede that there are probably few Japanese diabetic people with such a high BMI, unlike in Western populations.

The HRs for mortality from all causes, cardiovascular disease, cancer, and renal disease showed L-shaped relationships with BMI, which are compatible with the results from a previous study.^6^ Since cancer and cardiovascular disease are leading causes of death in Japan, the association between BMI and risk of all-cause mortality might be greatly affected by that between BMI and those diseases. Therefore, it stands to reason that, in countries where the leading cause of death is infectious disease, an obesity paradox might be observed in all-cause mortality.

There are several strengths to this study. First, the current study has a prospective design and a long duration of follow-up. In addition, setting cause-specific as well as all-cause mortality as endpoints was useful for detecting potential relationships associated with the obesity paradox.

Several limitations should be addressed. First, although the prognosis for diabetic people could be affected by glucose control, medications, complications of diabetes, and duration of diabetes, we have no information on glucose control and duration of diabetes. For example, patients with longer duration and worse control of diabetes might be likely to be leaner, and as a result, those with lower BMIs might have higher risks of mortality, as we found in the present study. Therefore, although we minimized the influence of major complications of diabetes (macro- and micro-vascular disease) on mortality by excluding diabetic people with cardiovascular disease and renal disease at baseline, we cannot completely exclude the possibility that BMI in the current study just reflects the severity of diabetes and the risks of mortality (ie, an inverse association). Second, we could not differentiate between different types of diabetes mellitus. However, since type 1 diabetes in adults is extremely rare in Japan, almost all cases among adults in Japan would be expected to be type 2.^19^

In conclusion, our observational study suggests that an obesity paradox in diabetic people may be observed with respect to mortality from infectious disease. However, it is still unknown whether or not the obesity paradox is observable in Asian diabetic people with BMI ≥40.0 kg/m^2^. Further investigation is needed to verify this supposition.

## ONLINE ONLY MATERIAL

Abstract in Japanese.
