# Supplementary material for: Association of Body Mass Index and Mortality in Japanese Diabetic Men and Women Based on Self-Reports: The Japan Collaborative Cohort (JACC) Study
Source: J Epidemiol. 2015 Aug 5;25(8):553–8. doi: 10.2188/jea.JE20150011 (PMC4517994; doi:10.2188/jea.JE20150011)
Supplement: Abstract in Japanese. [file je-25-553-s001.pdf]

自己申告糖尿病患者の body mass index と死亡リスクとの関係: JACC study からの報告

久保田　康彦[1]、磯　博康[1]、玉腰　暁子[2]、JACC Study Group

[1] 大阪大学大学院医学系研究科社会医学公衆衛生学教室

[2] 北海道大学大学院医学系研究科社会医学講座公衆衛生学分野

要約

背景：アジアの糖尿病患者における body mass index (BMI) と死亡との関連、特に、肥満パラドックス（BMI が大きくなるにつれ死亡リスクが低下する）が存在するかどうかに関しては解決していない。

方法：JACC study で自己申告による 40-79 歳の糖尿病患者のうち、循環器疾患、がん、腎臓病、および結核の既往のない 3,851 人（男性 2,115 人、女性 1,736 人）を 1998-1990 年から 2009 年まで追跡した。BMI で<20.0、20.0–22.9（reference）、23.0–24.9、および≥25.0kg/m2 の 4 群に分類し、Cox 比例ハザードモデルを用いて死亡リスクを比較した。

結果：54,707 人年の追跡期間中、全死亡 1,457 件、循環器死亡 445 件、がん死亡 421 件、腎不全死亡 43 件、および感染症死亡 148 件を認めた。全死亡、循環器死亡、がん死亡、および腎不全死亡のリスクと BMI との関連は L 字型を示した。一方、感染症死亡リスクと BMI と関連に関しては、BMI 20.0–22.9kg/m^2 に比べて、 BMI 23.0–24.9、≥25.0kg/m^2 の群は死亡リスクが低下する傾向にあった（肥満パラドックス）。多変量解析の結果、有意に感染症死亡リスクが低下していたのは、 BMI 23.0-24.9kg/m^2 でハザードリスク（95%信頼区間）=0.50 (0.31-0.81)、および BMI 25.0-27.4kg/m^2 で 0.51 (0.32-0.82)であった。喫煙状況および年齢で層化、また、早期死亡を除外した後も感染症死亡リスクと BMI との関連は同様であった。

結語：全死亡、循環器死亡、がん死亡、および腎不全死亡リスクと BMI との関連は L 字型を示したが、感染症死亡リスクと BMI との関連では肥満パラドックスを認めた。

キーワード：糖尿病、body mass index、死亡リスク、感染症、肥満パラドックス
